# European Network to Advance Best Practices and Technology on Medication Adherence: Mission Statement

**DOI:** 10.3389/fphar.2021.748702

**Published:** 2021-10-11

**Authors:** Job FM van Boven, Ioanna Tsiligianni, Ines Potočnjak, Jovan Mihajlović, Alexandra L. Dima, Urska Nabergoj Makovec, Tamás Ágh, Prezmyslaw Kardas, Cristina Mihaela Ghiciuc, Guenka Petrova, Noemi Bitterman, Fatjona Kamberi, Josip Culig, Björn Wettermark

**Affiliations:** ^1^ Department of Clinical Pharmacy and Pharmacology, Medication Adherence Expertise Center of the Northern Netherlands (MAECON), University Medical Center Groningen, University of Groningen, Groningen, Netherlands; ^2^ Department of Social Medicine, School of Medicine, University of Crete, Rethymnon, Greece; ^3^ Institute for Clinical Medical Research and Education, University Hospital Centre Sisters of Charity, Zagreb, Croatia; ^4^ Mihajlović Health Analytics, (MiHA), Novi Sad, Serbia; ^5^ University Medical Center Groningen, Groningen, Netherlands; ^6^ Medical Faculty, University of Novi Sad, Novi Sad, Serbia; ^7^ Research on Healthcare Performance (RESHAPE), INSERM U1290, University Claude Bernard Lyon 1, Lyon, France; ^8^ Faculty of Pharmacy, University of Ljubljana, Ljubljana, Slovenia; ^9^ Syreon Research Institute, Budapest, Hungary; ^10^ Medication Adherence Research Centre, Medical University of Lodz, Lodz, Poland; ^11^ Department of Morphofunctional Sciences II–Pharmacology, Clinical Pharmacology and Algesiology, School of Medicine, “Grigore T. Popa” University of Medicine and Pharmacy, Iasi, Romania; ^12^ Department of Social Pharmacy and Pharmacoeconomics, Faculty of Pharmacy, Medical University of Sofia, Sofia, Bulgaria; ^13^ Industrial Design, Technion, Israel Institute of Technology, Haifa, Israel; ^14^ Faculty of Health, Research Center of Public Health, University of Vlore “Ismail Qemali”, Vlore, Albania; ^15^ Pharmacoepidemiology Department, Andrija Stampar Teaching Institute of Public Health, Zagreb, Croatia; ^16^ Department of Pharmacy, Faculty of Pharmacy, Uppsala University, Uppsala, Sweden; ^17^ Faculty of Medicine, Vilnius University, Vilnius, Lithuania

**Keywords:** drug therapy, persistence, medication adherence, medication management, eHealth, medical technologies, COST Action 19132

## Abstract

Medication non-adherence is associated with almost 200,000 deaths annually and €80–125 billion in the European Union. Novel technological advances (smart pill bottles, digital inhalers and spacers, electronic pill blisters, e-injection pens, e-Health applications, big data) could help managing non-adherence. Healthcare professionals seem however inadequately informed about non-adherence, availability of technological solutions in daily practice is limited, and collaborative efforts to push forward their implementation are scarce. The European Network to Advance Best practices and technoLogy on medication adherencE (ENABLE, COST Action 19132) aims to 1) raise awareness of adherence enhancing solutions, 2) foster knowledge on medication adherence, 3) accelerate clinical application of novel technologies and 4) work collaboratively towards economically viable policy, and implementation of adherence enhancing technology across healthcare systems.

## Introduction

Thanks to several major achievements in drug discovery, we have seen an increasing number of patients surviving from diseases such as heart failure, stroke, asthma, and cancer as well as an improvement of the overall quality of life of the elderly population. Despite the availability of many effective medicines, it has been estimated that medication non-adherence, i.e., not taking medication as prescribed, is associated with almost 200,000 deaths annually (European Commission/MEDI-VOICE, 2011). Reasons for non-adherence can vary and can be categorized as either intelligent, erratic or unwitting, of which the former is also termed intentional and the latter unintentional (World Health Organization, 2003). From an economic perspective, non-adherence is responsible for €80–125 billion of potentially preventable direct (e.g., hospitalizations, waste of medication) and indirect (e.g., work productivity losses) costs in the European Union (EU) (European Commission/MEDI-VOICE, 2011). Unfortunately, medication non-adherence affects, depending on definitions used, up to 20–50% of patients who use medication for chronic diseases (World Health Organization, 2003). Moreover, in the last decades, there has been little improvement in adherence across and within the spectrum of multiple chronic diseases (Williams et al., 2008). Profound variance in adherence within- and between-countries, partly driven by factors such as low socio-economic status (Tøttenborg et al., 2016), suggests inequitable implementation of adherence supporting solutions. This is one of the reasons for which the World Health Organisation (WHO) has included medication adherence as a key indicator for quality of care (WHO, 2015). Of note, while adherence is an indicator for overall quality of care, it is not the ultimate goal of an intervention. In the end, the goal of treatment is to improve clinical, humanistic and economic outcomes with adherence only being a surrogate or intermediate endpoint. However, medication adherence is, together with several biological, pharmacokinetic and pharmacodynamic factors one of the key factors explaining inter-individual variability in clinical drug response (Harter and Peck, 1991; Van Boven et al., 2021). Without knowing the extent of medication adherence, one could assume that a drug is not working pharmacologically and therefore increase the dose or add another medicine. This not only puts the patient unnecessarily at risk for preventable side effects, but also increases the economic pressure on healthcare systems. As such, objective and granular insights into the extent of adherence is an essential element to understand the effect of medication and guide further treatment strategies. Importantly, access to proper data on medication adherence could also strengthen the capacity of–and help the conversation of the clinician with the patient about the issue of non-adherence which is currently still a major challenge for many physicians, nurses and pharmacists.

Assessment of adherence can be performed by a variety of techniques including patient self-report, questionnaires and bioanalytical methods (e.g., in blood, saliva, or urine) (Esteve-Romero et al., 2016). However, the former is often prone to social desirability or recall bias and the latter only provides a snapshot of adherence in the last one or 2 days and many require frequent invasive sampling. A further limitation of previous methods is that the correlation of these adherence measures with clinical outcomes is generally poor. For example, in a randomized controlled trial (RCT) testing a smartphone app to support adherence in patients with hypertension, there was an increase in the self-reported Morisky Medication Adherence Scale, yet blood pressure remained unchanged (Morawski et al., 2018). Recently developed digital technological advances (e.g., smart pill-boxes/packaging, digital inhalers, audio and vibration-based tracking devices, pill-tracers and e-injection pens, e-Health self-management applications, big data) seem promising with regards to generating more granular adherence data to support healthcare professionals and empower patients in detecting and managing non-adherence (Blakey et al., 2018; Zijp et al., 2020). The potential value of these remote monitoring solutions became increasingly evident during the COVID-19 pandemic (Taiwo and Ezugwu, 2020). However, the evidence underpinning these digital technologies is largely lacking. As such, the current application of these innovative technologies is still mainly limited to clinical trial settings, thereby not reaching healthcare professionals and patients in real-life practice. Even in clinical trial settings, outcomes do not always support scale-up of digital technologies. Indeed, contemporary trials, RCTs in particular, have highlighted the limitations of available technologies (Choudhry et al., 2017; Volpp et al., 2017; Morawski et al., 2018). Besides knowledge gaps regarding clinical efficacy, awareness of healthcare professionals on the availability and implementation of adherence enhancing technology is limited. Also, the technology is usually not embedded in a broader understanding of the reasons for suboptimal adherence. For example, sending reminders to patients being afraid of side effects is not going to increase their adherence. Furthermore, there is a lack of collaboration between key stakeholders to jointly work towards shared implementation goals (Clyne and McLachlan, 2015). Moreover, successful EU-wide implementation of innovative adherence enhancing technology in daily practice is further hampered by a lack of insight in the different European healthcare systems, reimbursement pathways and policy regulations that significantly differ from country to country (Khan and Socha-Dietrich, 2018).

Despite previous efforts such as the influential WHO 2003 report “Adherence to long-term therapies: Evidence for Action” (World Health Organization, 2003), stakeholders, including healthcare professionals, seem still inadequately informed about non-adherence and the availability of innovative technological solutions, and collaborative efforts to push forward their evidence base and implementation are scarce. To tackle this societal challenge and fuel policy, on October 20, 2020, the European Network to Advance Best practices and technoLogy on medication adherencE (ENABLE, COST Action 19132, https://www.cost.eu/actions/CA19132/) was launched. ENABLE is a 4-years COST Action funded by the European Commission, will run between 2020–2024, and brings together a network of 39 countries that 1) raise awareness of adherence enhancing solutions, 2) foster and extend multidisciplinary knowledge on medication adherence at patient, treatment and system levels, 3) accelerate translation of this knowledge from producers to useful clinical application and 4) work collaboratively towards economically viable policy and implementation of adherence enhancing technology across different European healthcare systems. This article provides an overview of ENABLE’s objectives, organization, working groups and outreach activities.

## Objectives

In order to tackle the challenges related to implementing solutions to reduce medication non-adherence, ENABLE has formulated three research coordination and three capacity building objectives as presented in Table 1.

**TABLE 1:** ENABLE’s research coordination and capacity building objectives.

ENABLE’s research coordination objectives
1. Benchmark, assess and raise awareness on current practices on tackling non-adherence in different healthcare settings across Europe
This entails the exchange of best practices, facilitators/barriers, experiences and expertise of different healthcare providers (GPs, specialists, pharmacists, nurses, health psychologists) and patients in dealing with non-adherence issues in daily life and practice
2. Accelerate the availability and implementation of adherence enhancing technologies across Europe
This entails the exchange (e.g., through webplatforms, vlogs, social media) of multidisciplinary knowledge and creates awareness among healthcare providers on the availability and potential of innovative adherence measurement methods and adherence enhancing technology (electronic monitoring, eHealth apps and tools) for clinical practice
3. Gather expert input for future market applications and implementation of cutting-edge adherence enhancing technology
To support timely patient access to technology, we facilitate knowledge exchange on cost-effectiveness, market access pathways, pricing, reimbursement and permanent integration of innovative adherence interventions in different European healthcare systems and clinical guidelines
**ENABLE’s capacity-building objectives**
1. Bringing together currently isolated stakeholders and building up a network
To achieve breakthroughs in the development of new process to advance the implementation of adherence enhancing technologies. This will be achieved by hosting meetings, workshops and conferences. Target stakeholders include clinicians, technologists, payers, and policy makers
2. Developing a platform and trans-national practice and policy community to exchange and foster knowledge
To achieve this, a web platform will be compiled and hosted which will include a repository of best practices and technologies that can enhance medication adherence, examples of successful implementation and patient access pathways. Per intervention, an independent assessment of its effectiveness, safety and cost-effectiveness will be provided by a panel of multidisciplinary experts not having a conflict of interest. Industry (e.g., pharmaceutical, IT) may propose an intervention to be included on the platform but cannot provide the aforementioned assessment
3. Translate and disseminate knowledge on technologies to specific target groups such as early stage researchers (being within 9 years of obtaining a PhD) and researchers from countries with less research capacity, as defined by the COST association
This will be achieved by hosting meetings and workshops, organizing Training Schools and opportunities for Short-Term Scientific Missions (STSMs). Through these STSMs, early stage researchers can learn from senior investigators and policy makers on the use and regulations of technology in other countries

## Organization

**FIGURE 1:**
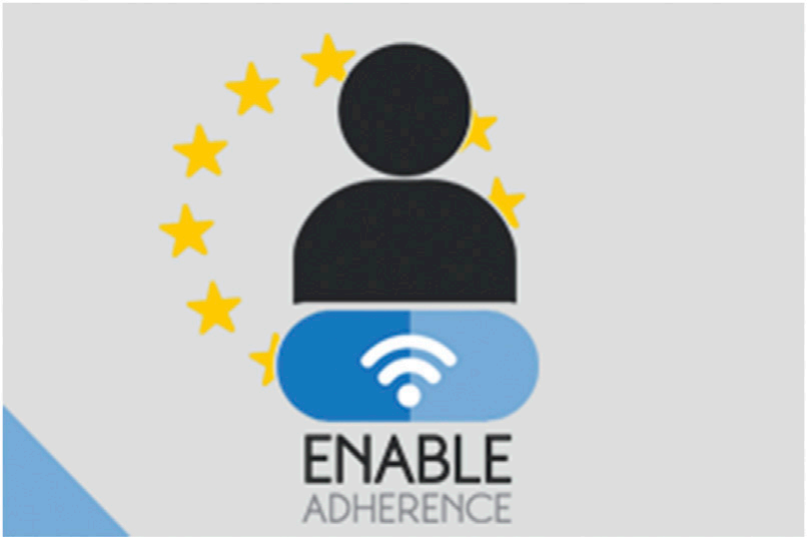
The Enable logo.

**FIGURE 2:**
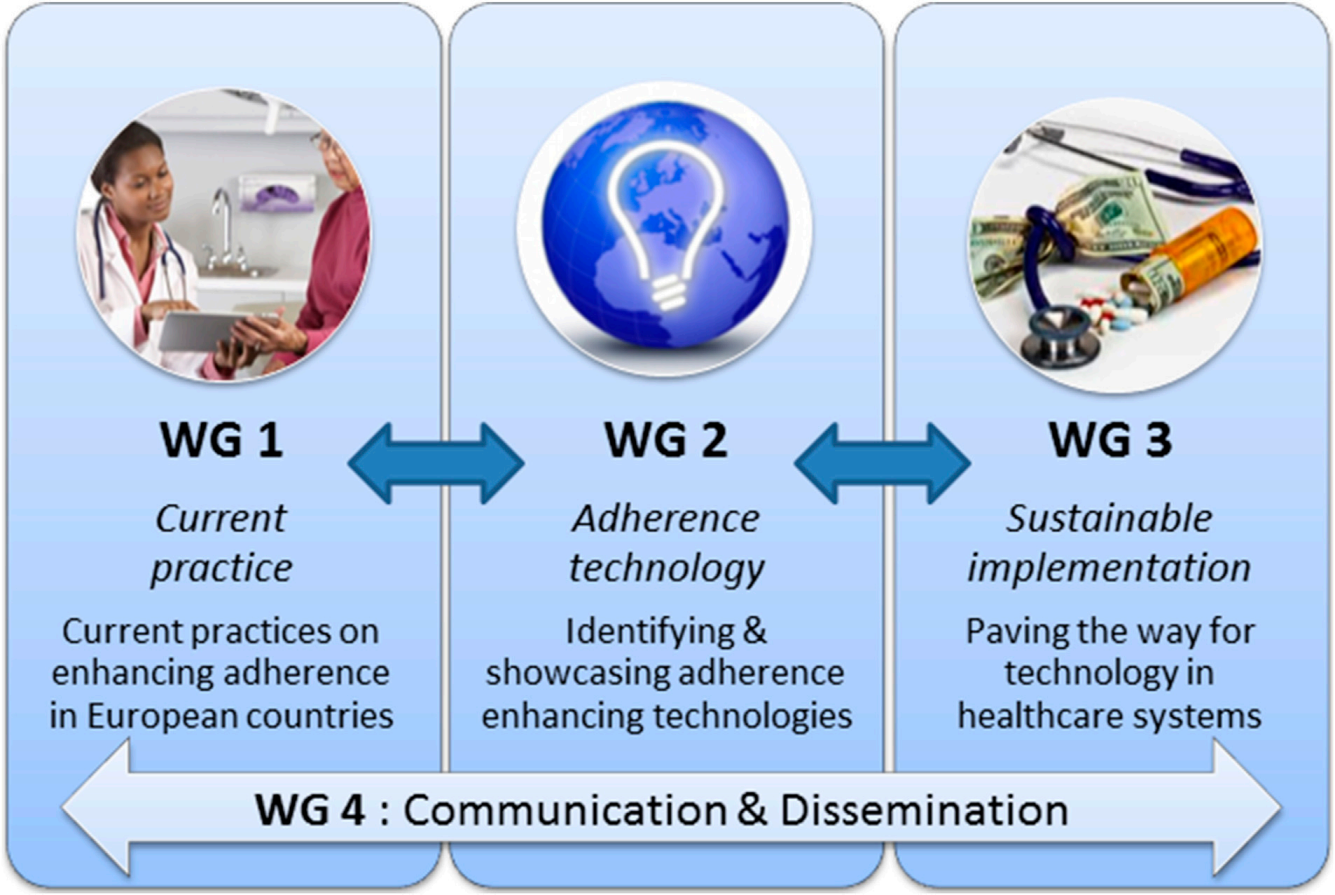
Structure of the ENABLE COST action.

In each of the 39 countries participating in ENABLE (Figure 1), multiple representatives from clinical practice are involved (physicians, pharmacists, psychologists, and nurses). Moreover, key people involved in clinical guideline development or people holding important positions in national or European clinical societies with the ability to influence and implement novel technologies are involved. In addition, patient associations, informal care associations, and regulators (registration authorities, payers, and health insurance policy makers) from each country will be targeted through the existing networks of ENABLE Action partners, but also by actively reaching out. Other main partners are the engineers that develop these technologies. Lastly, experts with specific expertise in e.g., communication and dissemination on a European level, such as partners involved in patient advocacy, will be asked to join the ENABLE Action. Stakeholder involvement will mainly be carried out through the networks of the country leads in the ENABLE Action, centrally coordinated by the responsible Working Group (Figure 2). To maximize exposure, materials will become available in local languages in a convenient way to take into account (health) literacy and cultural issues e.g. with infographics.

## Working Groups

ENABLE has produced a detailed work plan (Memorandum of Understanding: https://e-services.cost.eu/files/domain_files/CA/Action_CA19132/mou/CA19132-e.pdf) describing all the Working Group’s (WGs) tasks, activities, timeframes, and deliverables in order to assure coherent and effective work of the entire consortium, and timely achievement of its objectives. To fulfill the objectives, the ENABLE work plan includes the establishment of four inter-related WGs as depicted in Figure 2 and further described below.

### WG 1: Current Practices and Unmet Needs

WG1 focuses on the identification of means through which adherence is supported by healthcare professionals working across all levels of health care systems in the EU. In addition, best practices and successful adherence management strategies in settings within and outside European systems will be identified. The ultimate goal of the WG is to ascertain unmet needs to manage medication adherence in European settings, in order to raise awareness and stimulate further concrete actions building on state-of-the-art evidence and current best practices.

### WG 2: Adherence Enhancing Technologies

The aim of WG2 is to publicly showcase an extensive range of adherence enhancing technologies available in European countries on a dedicated repository on the COST ENABLE platform. To ensure the collection of rich information on a diverse range of adherence enhancing technologies on the COST ENABLE platform, project members will initiate and attend relevant networking activities (dissemination meetings, mobility activities) aiming to collect detailed descriptions of technologies, using a consensus-based template currently under development. They will also invite small and medium-sized enterprises (SMEs) and public organisations developing, providing and/or using such technologies to upload information (descriptions and multi-media) on the platform. A “digital networking market” will be organized during the project’s final conference to stimulate further research and implementation of medication support technologies.

### WG 3: Sustainable Implementation of Adherence Enhancing Technologies

The task of WG3 is to pave the way for adherence enhancing technology to be implemented in European health care settings. This will involve the sharing, collection, and dissemination of information on European healthcare systems (e.g., division of clinical tasks and responsibilities between primary care and secondary care as well as access to care), contact information of key stakeholder associations and reimbursement pathways/health technology assessment (HTA) guidelines for new adherence enhancing technologies.

### WG 4: Communication and Dissemination

The goal of WG4 is to disseminate and exploit the knowledge collected throughout the Action, develop and maintain the COST ENABLE webplatform (www.enableadherence.com), carry out the ENABLE communication plan, play a key role in the mobilization of stakeholders to participate and contribute to the action and the knowledge base. Lastly, WG4 will coordinate publication of the Action’s findings (Herdeiro et al., 2021; Kardas et al., 2021; Ágh et al., 2021).

## Outreach Activities

ENABLE uses different means to maximize outreach, visibility and involvement of stakeholders on the topic of medication adherence. We would like to stress that COST Actions are open environments with members from all COST countries being welcome to join and participate in the COST Action’s activities as provided below:

1) Maintain the ENABLE website (www.enableadherence.com) with aims, collaborators, and news/blogs/vlogs etc.;
2) Support establishment of national local “Medication adherence expertise centers,” “Medication Adherence Awareness Days” and stimulating adherence research;
3) Organize European conferences, e.g., to identify and actively invite key stakeholders and create roadmaps with unmet research and policy needs;
4) Organize stakeholder meetings linked to existing clinical/scientific conferences [e.g., The International Society for Medication Adherence (ESPACOMP), European Drug Utilisation Research Group (EuroDURG), disease focused conferences], to network and disseminate knowledge to non-specialist audiences;
(5) Provide Short-Term Scientific Missions (STSMs) for early stage and established researchers; these are short-term internships (usually lasting 1 week to 3 months) at European centers to learn more about adherence research and are funded by COST;
6) Provide Inclusiveness Target Country (ITC) grants to support investigators from lower resource countries (i.e., Albania, Bosnia and Herzegovina, Bulgaria, Croatia, Cyprus, Czech Republic, Estonia, Hungary, the North Republic of Macedonia, Latvia, Lithuania, Luxembourg, Malta, the Republic of Moldova, Montenegro, Poland, Portugal, Romania, Serbia, Slovakia, Slovenia, and Turkey) to attend international conferences and present their work related to medication adherence;
7) Organize Training Schools on medication adherence related topics;
8) Write editorials/call-for-actions in scientific, clinical, and policy-oriented conferences and journals, as well as on online network platforms such as LinkedIn, Twitter and ResearchGate; and
9) Distribute to national and international organizations that can influence the outcomes and build the change.

## Conclusion

Over the next 4 years, the ENABLE COST Action is expected to catalyze research, policy and implementation regarding medication adherence technologies and practices. The authors of this article invite everybody with interest in joining any of this Action’s activities to reach out and/or actively participate in ENABLE at either national of international level.

## Enable Collaborators

**Andrei Adrian Tica**, Romania; **Adriana Baban**, Romania; **Adriana E. Chis**, Ireland; **Alexandra Lelia Dima**, France; **Alexandru Corlăteanu**, Moldova; **Anna Bryndis Blöndal**, Iceland; **Anne Gerd Granås**, Norway; **Anthony Karageorgos**, Greece; **Bernard Vrijens**, Belgium; **Bettina S. Husebø**, Norway; **Bjorn Wettermark**, Sweden; **Catherine Goetzinger**, Luxembourg; **Christos Petrou**, Cyprus; **Çiğdem Gamze Özkan**, Turkey; **Cristina Mihaela Ghiciuc**, Romania; **Daisy Volmer**, Estonia; **Darinka Gjorgieva Ackova**, North Macedonia; **Dins Smits**, Latvia; **Dragana Drakul**, Bosnia and Herzegovina; **Elena Kkolou**, Cyprus; **Elín Ingibjorg Jacobsen**, Iceland; **Emma Aarnio**, Finland; **Enkeleda Sinaj**, Albania; **Enrica Menditto**, Italy; **Elena Kkolou**, Cyprus; **Eric Van Ganse**, France; **Esra Uslu**, Turkey; **Fatjona Kamberi**, Albania; **Fedor Lehocki**, Slovakia; **Francisca Leiva-Fernandez**, Spain; **Frederik Haupenthal**, Austria; **Freyja Jónsdóttir**, Iceland; **Gregor Bond**, Austria; **Guenka Petrova**, Bulgaria; **Hendrik Knoche**, Denmark; **Hilary Pinnock**, United Kingdom; **Horacio Gonzalez-Velez**, Ireland; **Indrė Trečiokienė**, Lithuania; **Ines Potočnjak**, Croatia; **Ioanna Chouvarda**, Greece; **Ioanna Tsiligianni**, Greece; **Isabel Leiva Gea**, Spain; **Isabelle Arnet**, Switzerland; **Ivana Tadić**, Serbia; **Ivett Jakab**, Hungary; **Jaime Correia de Sousa**, Portugal; **Jaime Espin Balbino**, Spain; **Jesper Kjærgaard**, Denmark; **Jiří Vlček**, Czech Republic; **Joao Gregorio**, Portugal; **Jolanta Gulbinovic**, Lithuania; **Josip Culig**, Croatia; **Jovan Mihajlović**, Serbia; **Juris Barzdins**, Latvia; **Katarina Smilkov**, North Macedonia; **Katerina Mala-Ladova**, Czech Republic; **Katharina Blankart**, Germany; **Konstantin Doberer**, Austria; **Laetitia Huiart**, Luxembourg; **Maja Ortner Hadžiabdić**, Croatia; **Manon Belhassen**, France; **Maria Cordina**, Malta; **Maria Teresa Herdeiro**, Portugal; **Marie Schneider**, Switzerland; **Martin Wawruch**, Slovakia; **Martina Bago**, Croatia; **Miriam Qvarnström**, Sweden; **Mitar Popovic**, Montenegro; **Mitja Kos**, Slovenia; **Natasa Duborija-Kovacevic**, Montenegro; **Noemi Bitterman**, Israel; **Omar S. Usmani**, United Kingdom; **Ott Laius**, Estonia; **Panagiotis Petrou**, Cyprus; **Paulo Félix Lamas**, Spain; **Paulo Moreira**, Portugal; **Petra Denig**, Netherlands; **Pilar Barnestein-Fonseca**, Spain; **Przemyslaw Kardas**, Poland; **Sabina De Geest**, Switzerland; **Seher Çakmak**, Turkey; **Stefan Bruno Velescu**, Romania; **Susanne Reventlow**, Denmark; **Tamás Ágh**, Hungary; **Urška Nabergoj Makovec**, Slovenia; **Valentina Marinkovic**, Serbia; **Valentina Orlando**, Italy; **Vered Shay**, Israel; **Vesna Vujic-Aleksic**, Bosnia and Herzegovina; **Vildan Mevsim**, Turkey; **Yasemin Cayir**, Turkey; **Yingqi Gu**, Ireland; **Zorana Kovacevic**, Serbia.
